# Supplementary material for: CDC20 Holds Novel Regulation Mechanism in RPA1 during Different Stages of DNA Damage to Induce Radio-Chemoresistance
Source: Int J Mol Sci. 2024 Aug 1;25(15):8383. doi: 10.3390/ijms25158383 (PMC11312485; doi:10.3390/ijms25158383)
Supplement: Supplementary file 1 [file ijms-25-08383-s001.zip › ijms-3060836-supplementary.pdf]

Table S1. **Primer sequences for RT-PCR analysis**

Gene	Forward primer	Reverse primer
CDC20	5'-AGACATTCACCCAGCATCAAG-3'	5'- CATCCACGGCACTCAGAC-3'
U6	5'- TCGCTTCGGCAGCACATAT -3'	5'- ATTTGCGTGTCATCCTTGC -3'

Table S2. **siRNA sequences of siRNA-NC and siRNA-CDC20**

Gene	Forward primer	Reverse primer
NC	5'-UUCUCCGAACGUGUCACGUTT-3'	5'-ACGUGACACGUUCGGAGAATT-3'
si-CDC20	5'-GGAAGACCUGCCGUUACAUTT-3'	5'-AUGUAACGGCAGGUCUUCCTT-3'

Table S3. **shRNA sequence of CDC20**

Gene	Sequence
shRNA-1	F: 5'- CCGGGCAGAAACGGCTTCGAAATATCTCGAGATATTTCGAAGCCGTTTCTGCTTTTTG-3'<br>R: 5'- AATTCAAAAAGCAGAAACGGCTTCGAAATATCTCGAGATATTTCGAAGCCGTTTCTGC-3'
shRNA-2	F: 5'- CCGGATGCGCCTGAAATCCGAAATGCTCGAGCATTTCGGATTTCAGGCGCATTTTTTG-3'<br>R: 5'- AATTCAAAAAATGCGCCTGAAATCCGAAATGCTCGAGCATTTCGGATTTCAGGCGCAT-3'
shRNA-3	F: 5'- CCGGCTAAGCTGGAACAGCTATATCCTCGAGGATATAGCTGTTCCAGCTTAGTTTTTG-3'<br>R: 5'- AATTCAAAAACTAAGCTGGAACAGCTATATCCTCGAGGATATAGCTGTTCCAGCTTAG-3'
